# Motivation and obstacles for weight management among young women – a qualitative study with a public health focus - the Tromsø study: *Fit Futures*

**DOI:** 10.1186/s12889-017-4321-9

**Published:** 2017-05-08

**Authors:** Anne-Sofie Sand, Nina Emaus, Olaug S. Lian

**Affiliations:** 1 Department of Clinical Research, University Hospital of North Norway, 9038 Tromsø, Norway; 2 Department of Health and Care Sciences, Faculty of Health Sciences, UiT The Arctic University of Norway, 9037 Tromsø, Norway; 3 Department of Community Medicine, Faculty of Health Sciences, UiT The Arctic University of Norway, 9037 Tromsø, Norway

**Keywords:** Young women, Overweight and obesity, Motivation for weight reduction, Environmental challenges, Lifestyle changes, Transition in life, Public health

## Abstract

**Background:**

Due to a worldwide increase in overweight and obesity, weight-management through lifestyle changes has become an important public health issue. Adolescents and young adults comprise a vulnerable group. The transition into adulthood represents a stage in life when establishing good lifestyle habits for the future is important. The aim of this study was to explore motivation and obstacles for weight reduction, weight maintenance and healthy lifestyle choices in young women.

**Methods:**

We conducted semi-structured in depth interviews with 12 young women, both overweight and normal weight, recruited from a school-based population survey. By the use of thematic analysis we searched the interview text for relevant meaning units that emerged as topics that illuminated our research questions.

**Results:**

A strong motivation for obtaining or keeping normal weight was clearly present among the participants. Independent of weight-group, the participants described increased levels of physical activity, better eating habits and regularity in daily life as desirable changes. Parents were described as important influencers regarding lifestyle habits. Several participants expressed a need for more information about healthy nutrition and eating. Their motivation for physical activity depended on a positive social setting and elements of joy. The participants described the transition into adulthood including moving out of their parents’ home and other structural changes in everyday life, as challenging. It affected their food choices and eating habits and other lifestyle issues. High costs of healthy food and sports activities were frequently mentioned among the obstacles they encountered.

**Conclusion:**

The results revealed an obvious motivation for lifestyle changes in individuals and environmental challenges for young women in the relevant stage of their life-course. There seems to be a need for health strategies that strengthens individuals’ capacity to overcome the environmental challenges in the transition to adulthood. This should include reliable and accessible health education/information regarding healthy nutrition, eating habits, food choices and preparation of meals. Structural initiatives such as easier access to affordable healthy food and less expensive opportunities for physical activity and sports should be considered.

**Electronic supplementary material:**

The online version of this article (doi:10.1186/s12889-017-4321-9) contains supplementary material, which is available to authorized users.

## Background

After decades dominated by infectious diseases, the worldwide increase in overweight and obesity [1], together with the shift towards a neo-liberal public health ideology [2], have made weight reduction and weight maintenance through lifestyle changes an important topic in public health policies. The main objective is to prevent non-communicable diseases by promoting healthy lifestyles. Most efforts to reverse the development towards increased body weight have been unsuccessful so far, on both a population level and an individual level [3, 4]. Still, individual responsibility is in the forefront when public health policies are discussed, while the responsibility of relevant societal institutions, such as the food industry and the popular press, is downplayed [5]. The debate on advantages or disadvantages of strategies on a high-risk, individual level or a population level in public health issues has been going on for several decades [6]. In recent years, counter movements such as the initiative “Health at every size” have occurred. Questions concerning the massive focus on overweight, health risk, and weight loss strategies have been raised [7]. Motivation for weight loss is not always present in people with overweight, and there is evidence suggesting that the tremendous focus on weight loss and weight loss strategies can be confusing and even unhealthy [7]. Furthermore, researchers from several academic disciplines are questioning some of the central claims about the existence of an obesity epidemic and the alleged relations between overweight and obesity and adverse health outcomes [8].

Despite this, prevention and treatment regarding overweight and obesity in the younger age-groups have become increasingly relevant in research and public health policies [9]. There has been little research on socio-cultural determinants of food habits, physically activity and body size perception [10]. Young adults comprise a particularly interesting group in relation to the topics of weight reduction and weight maintenance. They are in a crucial stage of their life-course, about to establish patterns of food practices, physical activity and lifestyle habits in general [11, 12]. Furthermore, young women are considered vulnerable regarding weight gain, weight maintenance and lifestyle issues [13]. Internal factors, such as physical and psychological changes are highly relevant in adolescents and young adults. Substantial environmental changes, such as moving out of the parental home and changing school or work environment including social and cultural influences are likely to affect their general living habits. These factors are, however, not well explored [13–15].

### Weight management strategies, why is it so difficult?

Previous studies have revealed how people hold complex and sophisticated theories about how to stay healthy. Health theories and ideas about health and disease have been influenced by prevailing ideologies and mediated by socio-structural circumstances. Furthermore, the medical view has been focusing on the absence of disease, whereas lay health believes tend to be more complex and nuanced [16]. The difficulties with finding effective solutions for weight reduction and weight maintenance remind us of the multifactorial complexity of all health-related issues [5], including the social stratifications and health inequalities [17]. Debates about the causes of and the solutions for the increasing rates of obesity have been going on for decades. There has been a focus on interventions in schools and workplaces, aiming at changing individuals’ lifestyle habits, and on environmental factors such as restricted advertising of unhealthy food [9]. In many countries, governments have introduced public policies and strategies regarding overweight and lifestyle issues. One example is “The Healthy People 2010 goal” from the US aiming at reducing the proportion of people with excess weight to that found in 1970 [9]. As such strategies proved to be unrealistic, initiatives aiming at prevention and collaboration with the food industry have turned up. One prominent example regarding prevention is the US White House Task Force on Childhood Obesity [3]. There are however many simplistic dichotomies in the field, such as treatment versus prevention and a predominance of top-down rather than bottom-up drivers of change. Top-down drivers are typically national or regional initiatives for spreading information about healthy lifestyle choices, while bottom-up drivers are initiated to create citizen engagement. In light of that, youth advocacy and online forums could prove to be important and effective contributors for obesity prevention [18].

Previous research has shown that when children and adolescents are trying to lose weight, the are many different reasons for this. Some studies have shown that social pressure and the wish to be accepted by peers are important reasons, while others report that desires for better appearance or better health are pivotal. Parents and family can be important influencers [19]. In adults, concerns about weight and shape are common in both people with normal weight and those who are overweight, and strategies on weight management are widespread [20].

Furthermore, media plays a pivotal role regarding health and lifestyle issues. The huge flow of information from different sources provides an opportunity to choose by personal preferences rather than reliability [21]. Young people are vulnerable to our appearance-oriented culture and the squeeze between cultural norms, health messages and their own perceptions [22]. The impact from social media and social comparison may be strongly connected to body dissatisfaction and negative self-esteem [23, 24].

However, the young should not be regarded as a homogenous group, and fluctuating subgroups are probably numerous [21]. Hence, the picture of underlying conditions regarding weight issues is complex. Discrepancies between intentions and actual behaviour must be seen in the light of these aspects.

Thus, the rationale for this study was to expand our knowledge within this complexity. We did this by blending individual and environmental perspectives in the presentation and discussion of the results from interviews with young women at the entrance to their adult life.

### Aim

The aim of the present study was to explore how motivational and environmental factors support and obstruct weight reduction and weight balance among young adult women. Our main research question was: What are the most important motivational and environmental factors that support or obstruct lifestyle changes and weight reductions or weight maintenance among young women? We discuss our findings in relation to relevant public health perspectives on weight issues in the young population.

## Methods

### Study design

The study was conducted using a qualitative research design and a phenomenological, hermeneutic approach. To get access to peoples’ experiences and illuminate their life-world, this methodological approach is suitable [25, 26]. The first author performed semi- structured life-world interviews as described by Kvale & Brinkmann [27]. In such research interviews, the interviewer defines the situation by introducing the interview topics and critically follows up the participants’ answers to the questions. The purpose of the interview is to obtain descriptions of the participants’ life-world in order to interpret the meaning of the described phenomena [27]. Initially, we asked the participants about their opinion concerning the attention and focus on overweight and lifestyle issues in the media, at school, at home and in society in general. With the use of an interview guide, we secured that the following topics were covered: food and nutrition, physical activity, sleeping habits, stress in everyday life, media influences, and lifestyle as a concept (Additional file 1).

The topics were chosen after thorough considerations of both well known and more recently emerging relevant factors for lifestyle habits and choices.

### Participants

The included women represented two different weight groups. One group was moderately overweight or slightly obese, with a Body Mass Index (BMI) range from 27.0 to 32.9 kg/m^2^ and the other group was within the normal weight range with a BMI range from 18.5 to 24.9 kg/m^2^ [28]. The reason for inviting two different groups regarding BMI-category was that we wanted to explore variations in perceptions in order to gain a broad picture. Striving for weight management is not exclusively experienced by persons who are overweight. We did not include participants close to or with severe underweight or severe obesity, as these participants were more likely to be under medical treatment. No participants were younger than 18 years, and participants older than 21 years were excluded. Only young women were included since weight concerns have been strongly connected to women [13, 23]. However, this picture seems to be changing according to recent research as body size concerns have shown to be increasing in male adolescents and young men [22, 29]. Thus, the reason for recruiting women only was based on an intention to focus the study.

The included participants were recruited from the second wave of a school-based population survey, Fit Futures 2 – part of the Tromsø study, performed in 2012/2013. To that survey, a total of 1130 students were invited, and 870 participated, giving an attendance rate of 77%. All the examinations, including weight, height and collection of self-reported data, were performed in one visit at the Clinical Research Unit at the University Hospital of North Norway. A comprehensive digital questionnaire covering multiple topics was developed for the study and performed on available laptops. The questionnaire included background data about family, school and living conditions, as well as questions about lifestyle habits and self-perceived health. The average BMI was 22.9 kg/m^2^ in women and 23.4 kg/m^2^ in men [30]. The proportion of participants with overweight and obesity according to the BMI classification groups as defined by The World Health Organization (WHO) [28], was 20.3% in women and 28.3% in men [30].

For the present qualitative study, the recruitment procedure was performed by staff from the Fit Futures study administration. Letters with invitation to participate were sent to eligible participants. Those who wanted to attend answered the invitation by mail, e-mail or phone. The response rate was disappointingly low, only 10% in the overweight group and 15% in the normal weight group. Reasons for this could be tiredness after attending surveys, or practical obstacles after finishing school and moving away. Another option is that we had underestimated the sensitivity of the subject in question.

### Analysis

The first author performed verbatim transcriptions of the interviews. By means of thematic analysis, we identified and reported patterns within the data, as described by Lindseth & Norberg [31] and Braun & Clarke [32]. Thematic analysis is a method for identifying, analysing and reporting patterns or themes in the data. More specifically, it organizes and describes the data-set while emphasizing context and integrating both manifest and latent contents in a non-linear analysis process [32, 33]. The sensitivity for context in our study made this method suitable.

Furthermore, even though we had participants from two different weight groups, comparisons between them were not emphasized in the interview nor in the analysis, making thematic analysis relevant [31, 32]. Contrary to a content analysis, a single comment was considered as important as those that were repeated [34].

The first author searched the texts for meaning units, as sentences or paragraphs containing information significant for the research topic [31]. After discussions with the co-authors, the first author subsequently suggested and developed themes through condensation of the meaning units. We identified themes by an interpretive process, scrutinizing all the meaning units from one interview and combining them with units and themes from the other interviews [35]. We chose themes on how they captured essential factors in relation to the research question [31, 33]. The analysis was done as a reflexive process, with re-reading and coding for many potential themes [32, 34]. Throughout the study, from planning to publication, we kept an audit trail documenting all important notes and amendments made on the way.

## Results

The interviews were performed at the Clinical Research Unit at the University Hospital of North Norway, shortly after the completion of the Fit Futures 2 survey. The interviews were audiotape recorded and the duration of each interview was between 43 and 81 min, with a mean duration of 56 min. All the participants were living in the coastal township of Tromsø, a university city in the Northern part of Norway with an overall ethnical, religious and cultural homogenous population. The number of inhabitants is approximately 72.000, with the majority living in the town centre. The participants’ characteristics are shown in Table 1.

**Table 1:** Participants’ characteristics

Age at interviews, all subjects (years)	18-19
Overweight group (n)	6
Living with parents (n)	3
Moved out (n)	3
Upper Secondary School (n)	3
University (n)	0
Work/apprenticeship (n)	3
Normal weight group (n)	6
Living with parents (n)	4
Moved out (n)	2
Upper Secondary School (n)	0
University (n)	3
Work/apprenticeship (n)	3

The results in the present study are mainly based on experiences of the young women in the overweight group. The data from the normal weight participants are used mainly as complementary data. The comparative element of the recruitment strategy was utilized in a previous paper [36]. For the present study, comparison was less important. Participants with normal weight provided useful information as weight concerns seems to be widespread in all weight groups in young adults. In addition, prevention of unhealthy weight gain is highly important in the younger age groups.

As already mentioned, blending individual and environmental factors in our search for supporting and obstructing factors is a core element in our study as shown in Table 2. Both dichotomies are important while we structure, present and discuss our empirical data.

**Table 2:** How environmental and individual factors support or obstruct weight reduction/maintenance and positive lifestyle habits

1. Individual – support	2. Individual – obstruct
• Wishes for becoming slimmer • Wishes for becoming healthier • Feeling better with better lifestyle habits • Ability to change gives increased self confidence	• Uncertainty about which food is healthy, information gap • Considering weight and body shape negatively can be demotivating regarding lifestyle changes (mixed feelings) • Aware of ones own bad habits but not willing to change
3. Environmental – support	4. Environmental – obstruct
• Support from parents • The joy and social aspects of being physically active • Levels of activity and fitness influenced by community and identity • Structure and responsibilities in everyday life make changes for better lifestyle habits easier	• Practical and time limiting factors for good habits regarding nutrition and activity when moving out of the family home • High costs as obstacles for buying healthy food and participating in sports activities • Stress and sleeping problems connected to education and life-course dependent changes • The organization of physical activity in school experienced as demotivating by some

### Individual factors – Support

All the participants in the overweight group explicitly expressed sincere wishes and aspirations for weight reduction and positive lifestyle changes during the interviews. However, some participants described this as more important than others. Ambivalence and contradictions in the way these young women reflected on their body weight and issues connected to lifestyle, health and well-being, were visible in the data:

I have *always* wanted to become slim, and I still do.

(Participant 1, overweight group)

I wouldn’t mind being thinner but I don’t have any problems with my appearance…I exercise a couple of times a week and I eat normally.

(Participant 2, overweight group)

In the latter quote, the participant made it clear that healthy lifestyle habits were more important for her than the weight issue. Several participants mentioned positive expectations of and experiences from accomplishing lifestyle changes independent of just losing weight:

I have better attitudes regarding food now than I used to have….

and I am generally happier…I eat less and I eat healthier food.

(Participant 1, overweight group)

I really do (wish to become more active), not so much for improving my appearance but because I *do* want to live healthier.

(Participant 3, overweight group)

Furthermore, some participants clearly expressed negative feelings when talking about how they experienced their overweight:

I consider my overweight every morning when I get dressed, wondering what to wear.

(Participant 4, overweight group)

It *is* a fact that people who are overweight have lower self-confidence.

(Participant 3, overweight group)

Most participants described physical activity as genuinely joyful pursuits, but highly depending on social settings. They had good childhood memories from experiences like participating in team sports or hikes with family and friends.

One participant in the normal weight group described how physical inactivity and subsequently weight gain temporarily made her feel uncomfortable and even depressed. Eventually, she managed to increase her level of activity again and described it like this:

When I started exercising again, it helped insanely.

(Participant 11, normal weight group)

She described how resuming her high level of activity felt like a big relief, making her feel a lot better in all regards.

One participant from the overweight group described how she had actually managed to change her habits:

I used to have bad habits, I had maybe one meal each day and that could be really big. I have changed my eating habits, now I have three or four meals each day …and I have stopped eating candy and drinking sweet drinks every day…and I am really proud of myself…I do not have the cravings for sweets anymore.

(Participant 2, overweight group)

This participant obviously experienced increased confidence and pride regarding her own ability to improve her lifestyle habits.

### Individual factors - obstruct

Regarding healthy nutrition and good eating habits, women in both weight groups felt that they needed more information and more knowledge. Most of them could not remember learning much about this in school. There seemed to be considerable confusion about what kind of food is actually healthy, as these participants in the overweight group stated:

I think it’s difficult to know what is healthy…I read and hear a lot about it, but what is actually healthy? There is no point in telling us to “eat healthy!” when we don’t know what kind of food that is healthy.

(Participant 6, overweight group)

In spite of outspoken lack of knowledge, one participant said that she was highly aware of her own unhealthy eating habits:

It is a bit funny…I do have bad eating habits…not that I eat junk food, but I only have one real meal during the day…I’m not hungry in the morning, I don’t eat while I’m at school, and when I get home for dinner I eat a lot and I don’t get hungry again after that*.*

(Participant 3, overweight group)

It was quite common for some participants to skip ordinary meals, and some were not willing to sacrifice the treat of eating sweets and snacks:

It gets so boring (when asked about what’s stopping her from eating healthier) I love eating sweets! It’s so nice to sit down with the girls and have sweets and candy.

(Participant 6, overweight group)

A need for more reliable information about good nutrition, better eating habits and healthy food were present in both weight groups. Several participants mentioned Internet blogs as an important source of information about food and fitness. However, they questioned the reliability of this information:

There is a lot of nonsense (when discussing blogs) ...or even bullshit, if I may say so.

(Participant 6, overweight group)

This participant described how social media made her feel like being in two different worlds; the media world and “the real one”.

Participants in the normal weight group also expressed their scepticism regarding blogs:

Some blogs are about exercise and could be useful…. but people should be aware of the fact that some of the bloggers really don’t have a clue.

(Participant 09, normal weight group)

Some bloggers can influence people to eat healthier…but I do think that people should make their own judgements about what is best for themselves.

(Participant 11, normal weight group)

However, some highlighted the positive effects of blogs regarding lifestyle:

The bloggers are real people, and they all have their niches and themes, on exercise or food…and some provide nice recipes and a focus on healthy eating.

(Participant 12, normal weight group)

The data indicated insecurity and an obvious ambivalence among the participants regarding messages from the media, in particular Internet blogs regarding.

healthy food choices. The lack of reliable sources of information was obvious.

Regarding obstacles for physical activity, one participant in the overweight group described experiences from former sessions of Personal Exercise (PE) in school and the situation in the dressing room:

I almost felt embarrassed…when you’re standing in the dressing room and you know that you have gained weight and you’re not comfortable….it’s not cool to get undressed, or even worse, to go in the shower.

(Participant 4, overweight group)

In this way, the participant described her feelings about revealing her body shape in front of her classmates. She felt uncomfortable and almost humiliated, and described this as an obstacle for participating in physical activity.

Another participant in the overweight group described how a family member’s actions previously had destroyed her motivation for weight-loss:

My weight was measured every week (by a family member) and I grew very frustrated…I ended up rebelling when I reached secondary school.

(Participant 5, overweight group)

Despite her rebellion and mixed feelings, she made it clear that weight reduction was something she really desired. It is somewhat hard to interpret if these negative feelings about being overweight is an incentive for lifestyle changes or not. She was clearly motivated, but there were also signs of frustration and opposition revealing a more negative attitude for making the necessary effort.

### Environmental factors – Support

Nearly all participants in both weight-groups described their parents as supportive motivators regarding overweight and lifestyle matters. Those who were still living with their parents (Table 1) described this as good for achieving or maintaining a healthy lifestyle. When asked about the most important influencers for a healthy lifestyle, one of the participants in the overweight group replied:

I would say my parents…they influence me in a positive way, things could have been a lot worse without their education and knowledge on lifestyle issues.

(Participant 3, overweight group)

Several participants mentioned family rules and parent supervision as important concerning sleeping habits through adolescence, as these expressions demonstrate:

I was never allowed to sleep all morning, so I have always kept my hours.

(Participant 11, normal weight group)

My parents closed down the internet access in the evenings.

(Participant 12, normal weight group)

The participants mentioned many reasons for being physically active. Those who were exercising on a regular basis highlighted the pleasure and the social aspects of these activities, as this participant from the overweight group:

I loved being part of a team (about being active as a soccer player), we had so much fun. I made a big effort in PE at school because this was where I could easily achieve good results/marks.

(Participant 6, overweight group)

One participant from the normal weight-group emphasized the joy and the support of peers:

It is a focus on the ideal body…we exercise because it is fun, not because we have to…it is a good circle, we keep each other going …before it was important to be lean, now it’s important to be strong…there are many things you are supposed to be…just not overweight.

(Participant 10, normal weight group)

This participant made a point of being lean or fit as a contrast to being overweight. Fitness and the pursuit of looking strong was a recurrent topic, especially in the normal weight group.

Some participants also said it was easier to improve and maintain structure in everyday life when they were employed in a job compared to being a student. As expressed by this participant in the overweight group:

For me, weight reduction became easier with the everyday routines that comes with a regular job.

(Participant 4, overweight group)

This view was confirmed by a participant in the normal weight group:

I found it easier to maintain structure and regularity after I finished school and started to work full time. Work is more important than school. I just can’t oversleep.

(Participant 8, normal weight group)

These participants felt an increased sense of responsibility and reliability when they moved from upper secondary school to the role of employees (Table 1). Being physically active on a high level was also connected to better sleeping patterns, as described by this normal weight participant:

I go to bed early each night, around 9 o’clock. I need a lot of sleep.

It’s kind of boring but I just have to…I do not perform well if I’m sleep deprived.

(Participant 7, normal weight group)

### Environmental factors – Obstruct

While parents’ influence was highlighted as good for healthy lifestyle habits, moving out seemed to involve challenges (Table 1). Some of the more practical and environmental aspects of the transition into adulthood were expressed as major obstacles in connection to lifestyle choices. The participants frequently mentioned moving away from the parental home, new school or work settings, and general stress as challenges regarding food and exercise habits.

The participants relied on their parents’ efforts to prepare and serve them healthy food, and said it could be challenging to live up to these standards when they moved out:

Some of my friends who are living on their own are telling me that they don’t have hot meals every day…that sounds awful, sandwiches for dinner! …but I am sure I would do the same in their situation.

(Participant 10, normal weight)

This participant in the normal weight group was almost filled with dread when thinking about the day she had to prepare her own meals.

One participant in the overweight group simply said:

When I lived with my mother we had decent food, like fish and chicken, when you share kitchen with 5 other people it’s easier to just grab a frozen pizza.

(Participant 6, overweight group)

Some of the participants had the experience of moving away from their parental home when starting upper secondary school at the age of 16, and one participant in the overweight group described the changes involved like this:

I used to be lean, I did a lot of exercise, had a good rhythm in life…then school started and I fell ill with an infection, lacked the energy for exercise…had a long way to travel to school…did not cook for myself.

(Participant 4, overweight group)

Furthermore, she described how this initiated a longer period of weight gain, mood changes and even depression throughout upper secondary school.

Participants in both groups described practical barriers that occurred after moving away from home, particularly in relation to healthy food choices, high costs of healthy food, lack of time to prepare healthy meals and shared kitchen facilities. Some women stated that they would not have been able to afford to attend gyms or other sport facilities if they had not been economically supported by their parents, especially after moving out.

As mentioned before, most of the participants had good memories of being physically active durng childhood.

active during childhood. However, some had quite different stories to tell when it came to PE lessons in upper secondary school, especially in the overweight group:

They (the teachers) evaluate the result and not the effort…and I found that extremely demotivating, it was just a bother...PE lessons should be fun!

(Participant 2, overweight group)

The focus on results and achieving good marks was an important aspect, and they described this as problematic and even unfair and discriminating. The perception of a singular focus on results was supported by some of the participants in the normal weight group:

There was a singular focus on achievements, I just found it demotivating!

(Participant 12, normal weight group).

However, we found wide variations in experiences from PE lessons, with descriptions ranging from “great fun” to “extremely demotivating”. Enthusiasm towards physical activity was not limited to the normal weight group. Importantly, all the participants in both groups described physical activity as an important source for well-being. The participants had various reasons for being physically active, and found motivation from different sources.

Descriptions of the gym as a social meeting point or the joy of being part of a sports team were recurrent findings in both weight groups. The social aspect of different kinds of physical activity was obviously highly valued. The venues for or opportunities to these experiences were, however, note easily found by all participants.

All participants described finishing upper secondary school as a major transition in life. One participant in the normal weight group described it as a difficult step with a lot of expectations and surprises:

When I started at University…I found myself in an unknown environment,I met new people all the time…I felt exhausted…it was awful! I did not exercise for 3 months, I really felt the stress and I gained weight….I became really depressed.

(Participant 11, normal weight group)

By the time of the interview, she felt that she had regained balance, but she described the experience as a bit of a shock. The seriousness and the pressure of achieving good results at school, getting a good education and consequently enter new environments were almost too much, as expressed by another participant in the same group:

You feel the pressure in a lot of ways…to achieve good results at school without being regarded as a nerd…to find friends…to find yourself.

(Participant 12, normal weight group)

As these expressions reveal, stress in terms of imbalance between expectations and experiences was a problem, especially in relation to school/work situations and the general structure of life. Notably, all the participants in the overweight group mentioned sleeping problems, often described as connected to a lack of structure in their everyday life. One participant in the overweight group described using the computer as a reason for staying up late:

It feels so comfortable to just sit there with your laptop instead of going to sleep…sometimes I have about 3-4 hours of sleep during night and I have to take a nap after school…it becomes a viscous circle.

(Participant 5, overweight group)

Another participant in the same group described sleeping problems in connection with a temporary dropout from school:

I slept during the day and stayed up most of the night…and I had to struggle to change that pattern…eventually I had to ask for sleeping medication.

(Participant 2, overweight group)

Environmental obstacles such as new living conditions, stress in connection to new school settings, need for more reliable information and better skills regarding healthy food, as well as accessible and affordable ways of being physically active, were prominent in our results. We will now discuss our findings in the light of the tensions between motivational factors and these obstacles.

## Discussion

The aim of the study was to explore the presence and impact of motivation and external factors and their influence on weight management in young women. The results showed an obvious presence of motivation among the participants alongside experienced obstacles in their environment. Structuring the results around the individual/environmental and the supportive/obstructive dichotomies revealed interesting patterns in our data. First, two different themes emerged regarding individual matters and intrinsic motivation. One was the wish to be thinner and the difficulties of achieving this among the participants in the overweight group. The other was how lifestyle changes involving healthier food and eating habits, and being more physically active, were associated with increased well-being in both weight groups. This was not only dependent on wishes for weight reduction or weight balance, but also connected to general well-being and improved self-confidence (Table 2, square 1). However, the outspoken lack of reliable sources of information about healthy nutrition (Table 2, square 2) was described as an important obstacle on the individual level.

Support from parents was clearly present, and the participants seemed grateful for this (Table 2, square 3). They described their parents’ influence as generally important for maintaining a healthy lifestyle, especially through better nutrition and eating habits. Gortmaker et al. [9], highlights the responsibility and opportunities that parents and caregivers have in promoting lifelong healthy behaviours among children and adolescents. Several studies have shown that healthy eating like fruit and vegetable intake among schoolchildren and students are highly dependent on socio-economic factors, parental influence and home availability [37–39]. While our findings supported the impression of parents’ role as important, we also found substantial vulnerability, in spite of supportive families (Table 2, square 4). The participants described it as a challenge to live up to their parents’ standards of nutrition and preparing of meals. In addition, several participants stated that high costs of healthy food and sport activities were barriers for a healthy lifestyle because living on their own as students or working in low-income jobs forced them to keep their expenses down. These findings are in line with previous research, describing that the life stage of adolescence and early adulthood involves a variety of physical, cultural, social and psychological changes [22]. When the young leave home, they make their own choices on diet and other lifestyle habits. For some, and especially young women, this is an opportunity to rebel against their parents [11]. Several studies have found the transition phase from upper secondary school to university to be especially vulnerable regarding weight gain [13].

Our findings indicate that health education can be important in strategies regarding treatment and prevention of overweight in the young. There are several possible approaches. One way is to try to pressure, persuade or coerce, another way is to empower and strengthen the individuals’ capacity of taking control of their health [21]. For example, nutrition education has been shown to be most effective when it stimulates learning, skills and action, rather than just providing knowledge [40]. This is in line with findings from social and psychological sciences. Intrinsic motivation should not be underestimated when it comes to human behaviour. In our study, important elements connected to Self-Determination Theory, namely autonomy, mastery and purpose, as described by Ryan and Deci [41], were easily recognised in the participants’ descriptions of why lifestyle changes were desirable and how they could be achieved. Feelings of pride, joy, well-being, and higher confidence in connection to successful improvements of lifestyle habits were clearly visible in our data (Table 2, Square 1).

Regarding physical activity, the data reveal some interesting contradictions concerning motivation and obstacles, intentions and practice. The social aspect of physical activity and having fun was important (Table 2, square 3). On the other hand, we found negative experiences connected to PE in school, especially during late adolescence/upper secondary school (Table 2, square 2 and 4). These experiences were connected to the way the teachers evaluated performances and efforts, and were described as demotivating by participants from both weight groups. Furthermore, some experiences were connected to sensitivity regarding body size and shape, which in turn was described as a demotivating factor by participants from the overweight group.

Some participants mentioned healthiness and fitness as important topics. The focus on fitness and appearance in the media, and especially the social media, has been connected to unintended consequences, such as lower self-esteem, negative mood and body dissatisfaction in young women [23, 24]. However, these participants expressed that exercise and fitness were part of their identity. The huge variety in preferences, attitudes and degrees of motivation regarding physical activity in this gender and age group could prove to be a challenge for public health policies. Although not very visible in our data, we can not rule out that some people with overweight do not wish to lose weight. However, it is noteworthy that by participants in both weight groups, simply feeling well was presented as a major reason for exercising or being physically active (Table 1, square 1).

The participants frequently mentioned stress, mostly in connection with school and education, as a negative factor regarding the ability to improve lifestyle (Table 2, square 4). Furthermore, sleeping problems were reported by several participants, especially in the overweight group (Table 2, square 4). Poor sleep, in terms of both short and long sleep duration, is associated with weight gain and obesity [42]. According to The Norwegian Public Health Institute [43], the average sleep duration in Norwegian teenagers is 2 hours lower than recommended for this age group. According to our data, bad sleeping habits seemed to be linked to a general lack of routines in everyday life. Furthermore, our findings indicated that finishing school and starting to work seemed to help. As stated by some of the participants, work is regarded as more important than school when it comes to being punctual and performing your duties. There was an obvious and noteworthy contrast between negative stress in connection to school and positive experiences of responsibility and regularity when having a job.

### Strengths and limitations

A school based population study formed the basis for the sample, securing that age, living area and school progression were similar for all participants. The response rate was, however, disappointingly low. The small sample could constitute a limitation. However, purposive sampling and generating experiential data is not depending only on the number of participants. We regarded the data as rich and vivid enough to describe the aspects of life-world that was in our scope.

We did not include direct questions about body image concerns in the interview guide, and this is a potential limitation regarding the emerging themes from the data. Eating disorders are furthermore not commented in the paper, and this constitute a possible limitation of the study. It is well known that body weight, body image and eating disorders are closely connected, and should be considered together rather than separately when studying weight concerns [14, 44, 45].

The exclusion of male participants is another limitation. Body size concerns with following consequences regarding obesity prevention and health promotion are not exclusively experienced by women. However, this was a decision taken early during the planning of the study in order to have a focused paper. The suggested implications of the study should be viewed as tentative given the small sample and possible limitations regarding themes in the interview guide.

There is always a question of transferability regarding geographical locations and contingencies such as sociocultural aspects. Similar references and cultural values together with the development regarding media and modern communication systems should make the findings comparable across the Western world.

## Conclusions

To summarize, we found an obvious tension between individual, motivational factors and environmental factors when discussing weight management and lifestyle choices with young women. Our findings indicate that bottom up strategies such as health education aiming at strengthening individuals’ capacity of taking control of their lifestyle and health could prove to be useful. Making healthy food more accessible and affordable as well as offering low-cost alternatives for organized sports should be considered in public health strategies in the target group.

The findings of positive experiences of improved lifestyle habits when being employees and not students should be pursued in future research.

More research in bigger samples, and in both genders, is needed to pursue these findings and their relevance for public health strategies and necessary structural changes.

## Additional file

Additional file 1: Interview guide. Topics and questions used in the interviews, translated from Norwegian. (DOCX 99 kb)

## Abbreviations

BMI: Body mass index
PE: Personal exercise
UiT: University of Tromsø
WHO: World Health Organization

## References

[1] NCD Risk Factor Collaboration (NCD-RisC) (2016). Trends in adult body-mass index in 200 countries from 1975 to 2015: a pooled analysis of 1698 population-based measurements studies with 19.2 million participants. Lancet.

[2] LeBesco K (2011). Neoliberalism, public health, and the moral perils of fatness. Critical Public Health.

[3] Roberto CA, Swinburn B, Hawkes C, Huang TT-K, Costa SA, Ashe M (2015). Patchy progress on obesity prevention: emerging examples, entrenched barriers, and new thinking. Lancet.

[4] Ferraro ZM, Patterson S, Chaput JP (2015). Unhealthy weight control practices: culprits and clinical recommendations. Clin Med Insights Endocrinol Diabetes.

[5] Brown BJ, Baker S (2013). Responsible citizens. Individuals, Health and policy under Neoliberalism.

[6] Rose G (1985). Sick individuals and sick populations. Int J Epidemiol.

[7] Bacon L (2008). Health at every size. The surprising truth about your weight.

[8] Campos P, Saguy A, Ernsberger P, Oliver E, Gaesser G (2006). The epidemiology of overweight and obesity: public health crisis or moral panic?. Int J Epidemiol.

[9] Gortmaker SL, Swinburn BA, Levy D, Carter R, Mabry PL, Finegood DT (2011). Changing the future of obesity: science, policy, and action. Lancet.

[10] Swinburn BA, Sacks G, Hall KD, McPherson K, Finegood DT, Moodie ML (2011). The global obesity pandemic: shaped by global drivers and local environments. Lancet.

[11] Lupton D (1996). Food, the body and the self.

[12] Faw MH (2014). Young adults’ strategies for managing social support during weight-loss attempts. Qual Health Res.

[13] Wane S, van Uffelen JG, Brown W (2010). Determinants of weight gain in young women: a review of the literature. J Women’s Health (Larchmt).

[14] Neumark-Sztainer D, Paxton SJ, Hannan PJ, Haines J, Story M (2006). Does body Satisfation matter? Five-year longitudinal associations between body satisfaction and Health Behaviours in adolescent females and males. J Adolesc Health.

[15] Friscoe ML, Houle JN, Lippert AM (2013). Weight change and depression among US young women during the transition to adulthood. Am J Epidemiol.

[16] Nettleton S (2013). The sociology of Health and illness.

[17] Lahelma E. Health and Social Stratification. In: Cockerham WC, editor. The Blackwell Companion to Medical Sociology. Oxford: Blackwell Publishing Ltd; 2005. p. 64-93.

[18] Huang TT, Cawley JH, Ashe M, Costa SA, Frerichs LM, Zwicker L (2015). Mobilisation of public support for policy actions to prevent obesity. Lancet.

[19] Braden AL, Crow S, Boutelle K (2015). Child self-reported motivations for weight loss: impact of personal vs. social/familial motives on family-based behavioural weight loss treatment outcomes. Eat Weight Disord.

[20] Nissen NK, Holm L (2015). Literature review: perceptions and management of body size among normal weight and moderately overweight people. Obes Rev.

[21] Green J, Tones K (2010). Health promotion. Planning and strategies.

[22] Voelker DK, Reel JJ, Greenleaf C (2015). Weight status and body image perceptions in adolescents: current perspectives. Adolesc Health Med Ther.

[23] Tiggeman M, Zaccardo M. ”Exercise to be fit, not skinny”: the effect of fitspiration imagery on women’s body image. Body Image. 2015;15:61-7. 10.1016/j.bodyim.2015.06.003 26176993

[24] Fardouly J, Deidrichs PC, Vartanian LR, Halliwell E (2015). Social comparisons on the social media: the impact of Facebook on young women’s body image concerns and mood. Body Image.

[25] Dahlberg K, Drew N, Nyström M (2008). Reflective Lifeworld research.

[26] Polit DF, Beck CT (2008). Nursing research. Generating and assessing evidence for nursing practice.

[27] Kvale S, Brinkmann S (2009). Interviews. Learning the craft of qualitative research interviewing.

[28] WHO World Health Organization. Global database on Body Mass Index. www.assessmentpsychology.com/icbmi.htm. Accessed 2 May 2017.

[29] Mitchison D, Mond J (2015). Epidemiology of eating disorders, eating disordered behaviour, and body image disturbance in males: a narrative review. J Eat Disord.

[30] Sand AS, Furberg AS, Lian OS, Nielsen CS, Pettersen G, Winther A (2017). A cross-sectional study on the differences between measured, perceived and desired body size and the relation to self-perceived health in young adults. The Tromsø study: fit Futures. Scand J Publ Health.

[31] Lindseth A, Norberg A (2004). A phenomenological hermeneutical method for researching lived experience. Scand J Caring Sci.

[32] Braun V, Clarke V (2008). Using thematic analysis in psychology. Qualitative Res Psychol.

[33] Vaismoradi M, Turunen H, Bondas T (2013). Content analysis and thematic analysis: implications for conducting a qualitative descriptive study. Nurs Health Sci.

[34] Fereday J, Muir-Cochrane E (2006). Demonstrating rigor using thematic analysis: a hybrid approach of inductive and deductive coding and theme development. Int J Qualitative Meth.

[35] Morse JM (2015). Analytic strategies and sample size. Qual Health Res.

[36] Sand AS, Emaus N, Lian OS (2015). Overweight and obesity in young adult women: a matter of health or appearance? The Tromsø study: fit Futures. Int J Qual Stud Health Well-being.

[37] Nilsen SM, Krokstad S, Holmen TL, Westin S (2010). Adolescents’ health-related dietary patterns by parental socio-economic position, the Nord-Trøndelag Health study (HUNT). Eur J Pub Health.

[38] Wind M, te Welde SJ, Brug J, Sandvik C, Klepp KI (2010). Diet and direct interaction between environmental factors and fruit intake, mediation by psychosocial factors: the pro children study. Public Health Nutr.

[39] Sandvik C, Gjestad R, Samdal O, Brug J, Klepp KI (2010). Does socio-economic status moderate the associations between psychosocial predictors and fruit intake in schoolchildren? The pro children study. Health Educ Res.

[40] Hawkes C, Smith TG, Jewell J, Wardle J, Hammond RA, Friel S (2015). Smart food policies for obesity prevention. Lancet.

[41] Ryan RM, Deci EL (2000). Self-determination theory and the facilitation of intrinsic motivation, social development, and well-being. Am Psychol.

[42] Chaput JP, Després JP, Bouchard C, Tremblay A (2011). The association between short sleep duration and weight gain is dependent on disinhibited eating behaviour in adults. Sleep.

[43] Norwegian Institute of Public Health. Sleep problems in Norway. Public Health Report. 2014. https://www.fhi.no/en/mp/sleep-and-sleep-disorders/. Accessed 2 May 2017.

[44] Mitchison D, Hay P, Griffiths S, Murray SB, Bentley C, Gratwick-Sarll K, Harrison C, Mond J (2017). Disentangling body image: the relative associations of overvaluation, dissatisfaction, and preoccupation with psychological distress and eating disorder behaviors in male and female adolescents. Int J Eat Disord.

[45] Fredrickson J, Kremer P, Swinburn B, de Silva A, McCabe M (2015). Weight perception in overweight adolescents: associations with body change intentions, diet and physical activity. J Health Psychol.

