# Supplementary material for: Motivation and obstacles for weight management among young women – a qualitative study with a public health focus - the Tromsø study: Fit Futures
Source: BMC Public Health. 2017 May 8;17:417. doi: 10.1186/s12889-017-4321-9 (PMC5422962; doi:10.1186/s12889-017-4321-9)
Supplement: Additional file 1: — Interview guide. Topics and questions used in the interviews, translated from Norwegian. (DOCX 99 kb) [file 12889_2017_4321_MOESM1_ESM.docx]

**Interview guide**

Motivation and obstacles for weight management among young women – a qualitative study with a public health focus – The Tromsø Study: *Fit Futures*

| **Topic** | **Question** |
| --- | --- |
| Experience of overweight | General talk about the subject (as introduction). What is overweight (definition)?  The public focus on overweight issues - is it too much or too little?  Do you have any other thoughts on the subject? |
| Motivation for weight management | Is it important? If yes: how important? why is it important? If no: why is it not important? |
| Nutrition/food habits | What is healthy nutrition, in your opinion? How will you describe your food habits, as healthy or unhealthy?  Do you have regular meals? How do you eat during school/work days? Packed lunch or buy food in canteen or shop?  What is your opinion on young people and food habits? Do they care? What can be done to improve food habits in the young? |
| Physical activity | What are your habits and attitudes regarding physical activity? What is your impression of the habits and attitudes in the young, should they be more or less active? How can improvements be made? |
| Sleep | How many hours do you usually sleep during night? Do you sleep during day time?  Do you have any trouble regarding sleep? Do you think your peers have any trouble with their sleeping habits? What can be obstacles for good sleeping habits? What about internet use and social media as obstacles? |
| Lifestyle | What is your understanding of the subject (lifestyle)? Who and what influence your choice of lifestyle? |
| Media | Are body size ideals and lifestyle habits influenced by the media, including social media? |
| Participations in health surveys | Did participation in the health survey(s) have any influence on your perception of health or lifestyle? |
| Stress and coping | Is stress influencing your lifestyle? If yes: in what way? What about your peers? |
| Concluding question | Are there any other things of importance that you want to mention? |
